# Protein expression of S100A2 reveals it association with patient prognosis and immune infiltration profile in colorectal cancer

**DOI:** 10.7150/jca.83910

**Published:** 2023-06-19

**Authors:** Phimmada Hatthakarnkul, Aula Ammar, Kathryn A. F. Pennel, Leah Officer-Jones, Silvia Cusumano, Jean A. Quinn, Amna Ahmed Mohemmed Matly, Peter G. Alexander, Jennifer Hay, Ditte Andersen, Gerard Lynch, Hester C. van Wyk, Noori Maka, Donald C. McMillan, John Le Quesne, Chanitra Thuwajit, Joanne Edwards

**Affiliations:** 1 School of Cancer Sciences, University of Glasgow, Wolfson Wohl Cancer Research Centre, Garscube Estate, Glasgow, United Kingdom.; 2 Biomedical Science Program, Faculty of Medicine Siriraj Hospital, Mahidol University, Bangkok, Thailand.; 3 School of Medicine, University of Glasgow, Glasgow Royal Infirmary, Alexandria Parade, Glasgow, United Kingdom.; 4 Department of Immunology, Faculty of Medicine Siriraj Hospital, Mahidol University, Bangkok, Thailand.; 5 Glasgow Tissue Research Facility, University of Glasgow, Queen Elizabeth University Hospital, Glasgow, United Kingdom.; 6 Cancer Research UK Beatson Institute, Garscube Estate, Glasgow, United Kingdom.; 7 BioClavis Ltd, Glasgow, United Kingdom.

**Keywords:** S100A2, inflammatory, colorectal cancer

## Abstract

Purpose: Colorectal cancer (CRC) is the third most diagnosed cancer worldwide. Despite a well-established knowledge of tumour development, biomarkers to predict patient outcomes are still required. S100 calcium-binding protein A2 (S100A2) has been purposed as a potential marker in many types of cancer, however, the prognostic value of S100A2 in CRC is rarely reported.

Material and Methods: In this study, immunohistochemistry (IHC) was performed to identify the prognostic role of S100A2 protein expression in the tumour core of the tissue microarrays (TMAs) in colorectal cancer patients (n=787). Bulk RNA transcriptomic data was used to identify significant genes compared between low and high cytoplasmic S100A2 groups. Multiplex immunofluorescence (mIF) was performed to further study and confirm the immune infiltration in tumours with low and high cytoplasmic S100A2.

Results: Low cytoplasmic protein expression of S100A2 in the tumour core was associated with poor survival (HR 0.539, 95%CI 0.394-0.737, *P*<0.001) and other adverse tumour phenotypes. RNA transcriptomic analysis showed a gene significantly associated with the low cytoplasmic S100A2 group (*AKT3*, *TAGLN*, *MYLK, FGD6* and *ETFDH*), which correlated with tumour development and progression. GSEA analysis identifies the enriched anti-tumour and immune activity group of genes in high cytoplasmic S100A2. Additionally, mIF staining showed that high CD3+FOXP3+ and CD163+ inversely associated with low cytoplasmic S100A2 (*P*<0.001, *P*=0.009 respectively).

Conclusion: Our finding demonstrates a prognostic value of S100A2 together with the correlation with immune infiltration in CRC.

## Introduction

Colorectal cancer (CRC) is the fourth most diagnosed cancer in the UK and a major cause of worldwide cancer-related mortality. 1. Advances in surgery, chemotherapy and the screening program have dramatically improved patients' survival, however disease metastases remain a challenge for cancer treatment 2-4. Many approaches have been proposed in terms of the identification of novel prognostic markers in CRC, however, only a few have been translated to the clinic 5.

There is increasing evidence that the family of proteins S100, a conserved sub-family of elongation factor (EF)-hand type calcium-binding proteins, may have a prognostic role in tumour progression 6, 7. S100 calcium-binding protein A2 (S100A2) has been reported to function either as a tumour suppressor or promoter 8, 9. Although the function of this protein requires elucidation, S100A2 has been described as a potential predictive marker in various types of cancer 10-16. Despite its role in malignant disease, though only reported in a few studies, S100A2 was also involved in the development of inflammatory disease 17.

Immunotherapy has emerged as a treatment option as a result of recent publications suggesting a role for inflammation in CRC progression and metastasis 18, 19. S100 proteins have been shown to play an important role in inflammatory and autoimmune diseases 20. For example, the binding of S100A8/A9 to TLR4 could stimulate inflammation, cell proliferation and tumour development through NF-kB signalling 21, and its elevation was shown to mediate the effect of tumour necrosis factor- alpha (TNF-α) during chronic inflammation 22. Despite a few studies, S100A2 may be related to the regulation of different populations of immune cells 23. This study aims to identify the prognostic value of S100A2 and its association with infiltrating immune cells in CRC patients.

## Material and Methods

### Patient cohorts

A cohort of 787 patients with stage I-III CRC who had undergone surgical resection at Glasgow Royal Infirmary (Glasgow, UK) between 1997 and 2013 was included in immunohistochemistry (IHC) and multiplex immunofluorescence (mIF) analysis. The inclusion criteria of patients enrolled in this study are that if this was the first cancer with no preceding history, presented for the first time at the clinic and were excluded if they had co-morbidities. In addition, patients who died within 30 days of surgery, had emergency surgery or received neoadjuvant therapy were excluded. A previously constructed TMA was utilised for IHC and mIF experiments with three 0.6 mm cores per patient to account for tumour heterogeneity. Tumour staging was carried out using the 5^th^ Edition of the AJCC/UICC-TNM staging system by the time tissues were collected. Clinicopathological data were collected with a minimum of 5 years follow-up years post-resection.

### Western blot

Western blot analysis was conducted to demonstrate the specificity of antibodies used for IHC. S100A2 293T cell transient overexpressed lysate (H00006273-T01, Novus biologicals) was loaded into a 4-12% sodium dodecyl sulfate-polyacrylamide gel and separated by electrophoresis (SDS-PAGE). The protein was then transferred to a polyvinylidene fluoride (PVDF) membrane and blocked with 5% skimmed milk in Tris-buffered saline with Tween (TBST). The membrane was incubated with S100A2 antibody (PA5-31861, Thermofisher, 1:5000) and then with secondary antibody conjugated HRP (7074S, Cell signalling, 1:6000). Finally, the HRP signal was detected using PierceTM ECL Western (Thermo ScientificTM). The membrane was imaged using a Gel Doc instrument (G:Box Chemi XR5, Cambridge, UK). β-tubulin (ab21058, Abcam, 1:5000) was used as a loading control.

### Immunohistochemistry (IHC)

IHC was performed on a previously constructed TMA (n=787). Briefly, TMAs were dewaxed by immersion in Histoclear and rehydrated through a series of alcohols. Heat-induced antigen retrieval was performed in citrate buffer (pH6), after which the sections were incubated in 3% H_2_O_2_. Non-specific binding was blocked with 5% horse serum before overnight incubation with S100A2 antibody at 4^o^C (PA5-31861, 1:4000, Thermofisher). Staining was visualised using ImmPRESS and ImmPACT DAB (Vector Laboratories, SK4105). The tissue was counterstained using Haematoxylin Gill III (3801540E, Leica Biosystems) before being dehydrated and mounted using Pertex® (SEA-0100-00A, Histolab). Appropriate negative controls were included.

### Scoring method

Stained TMA sections were scanned using a Hamamatsu NanoZoomer (Welwyn Garden City, Hertfordshire, UK) at x20 magnification on NDP.view2 (version 2.8.24). The weighted histoscore was calculated for cytoplasmic S100A2 expression as follows: 0x not stained + 1x weakly stained + 2x moderately stained + 3x strongly stained. A range of scores from 0 to 300 was obtained for cytoplasmic staining. Manual histoscoring was employed to assess expression, 10% of cores were double-scored by an independent observer with the correlation coefficient >0.7 achieved.

### Transcriptomic analysis

Single tissue sections from CRC cohort (n=787) who had undergone resection for CRC were used for Templated Oligo-Sequencing (TempO-Seq) analysis using a Whole Transcriptome panel. Briefly, formalin-fixed paraffin-embedded (FFPE) tissue was deparaffinised prior to tissue digestion. The tissue lysate was combined with detector oligos which were annealed in immediate juxtaposition to each other on the targeted RNA template and ligated 24. Amplification of ligated oligos was performed using a unique primer set for each sample, introducing a sample-specific barcode and Illumina adaptors. Barcoded samples were pooled into a single library and run on an Illumina HiSeq 2500 High Output v4 flowcell. Sequencing reads were demultiplexed using BCL2FASTQ software (Illumina, USA). FASTQ files were aligned to the Human Whole Transcriptome v2.0 panel, which consists of 22,537 probes, using STAR 25. Up to two mismatches were allowed in the 50-nucleotide sequencing read.

### Gene Set Enrichment Analysis (GSEA) for S100A2

In this study, the normalised counts of TempO-Seq data (n=610) from DESeq2 were utilised and analysed through the GSEA program 26 (https://www.gsea-msigdb.org/gsea/msigdb/index.jsp). The molecular signature database (MSigDB) was used based on the comparison between tumours with low and high cytoplasmic S100A2 27. The enrichment pathways were determined based on the nominal P-value and false discovery rate (FDR).

### Multiplex immunofluorescence (mIF)

Two panels of antibodies were used to perform mIF on CRC TMAs (n=787). On panel 1, a fully automated mIF assay was developed on the Ventana Discovery Ultra autostainer platform (Roche Tissue Diagnostics, software version RUO Discovery Universal V21.00.0019). Staining was performed on 4 µm thick sections of previously constructed TMAs with the optimised antibodies (Table S1). A negative control slide was used on each staining run to rule out non-specific staining. Whole slide images were captured at 10x magnification using the PhenoImager HT multispectral slide scanner (Akoya Biosciences V1.0.13), TMA maps were applied using Phenochart software (Akoya Biosciences V1.1.0), and core images were captured at 20x magnification. Core images were spectrally unmixed using Inform software (Akoya Biosciences, software version 2.5.1).

mIF panel 2 was stained using an autostainer (Thermofisher) with optimised antibodies (Table S1). The slides were scanned by NanoZoomer S60 digital slide scanner (Hamamatsu, USA) with 20x magnification. TMA maps were applied for further analysis. Visiopharm (version 2021.02.5.10297), a digital precision pathology software, was used to perform the analysis. The percent positive cells of total cells detected for each marker were calculated (Figure S1).

### Statistical analysis

The TempO-Seq data was analysed in R studio using DESeq2 packages. The differential gene expression between the patients with low and high cytoplasmic S100A2, was analysed for statistical significance using the Wald test.

Maxstat and survminer packages were utilised to determine optimal thresholds for high and low expression groups for weighted histoscores and counts from multiplex staining. The statistical analysis was performed in IBM SPSS Statistic Version 27.0. Pearson's χ2 test assessed the relationship between cytoplasmic S100A2 expression and clinicopathological features. The likelihood ratio and Mann-Whitney U test were used when required. Patient survival was assessed by Kaplan-Meier analysis and log-rank to test the significance. Univariate and multivariate Cox hazard regression was performed to estimate the hazard ratio (HR) for cancer-specific survival (CSS) and identify the significant prognostic factors in CRC patients. For the association with immune cells, bar charts were plotted using using GraphPad Prism version 8 (GraphPad Software Inc.). In this study, a p-value or nominal p ≤ 0.05 with FDR < 0.25 was considered statistically significant.

## Results

### Clinicopathological parameters of 787 CRC cohort

To further investigate the role of S100A2 at the protein level, the TMA from the GRI CRC cohort (n=787) was utilised. This was reduced from 787 to 644 CRC patients after exclusions were applied (Figure S2). 205 (32%) patients were under 65, 208 (32%) were between 65-74 and 231 (36%) were over 75 years of age, with n=353 males and n=291 females. The median follow-up was 91 months. 644 patients had valid scores for S100A2 tumour cytoplasmic staining (Table S2).

### Immunohistochemistry of S100A2 in CRC TMAs

Protein expression of S100A2 was determined by IHC in TMAs to investigate its role in CRC. Firstly, antibody specificity was performed using western blot. A single band was observed by western blot using a commercial 293T cell lysate overexpressed with S100A2 and a faint band was observed for HCT116 cell line lysate, known to express S100A2 at low levels, and a stronger band was shown for MDA-MB-231 breast cancer cell line lysate, known to express S100A2 at higher levels. Expression of lysates was identified using the cancer dependency map (DepMap; https://depmap.org/) (Figure S3).

**Figure 1:**
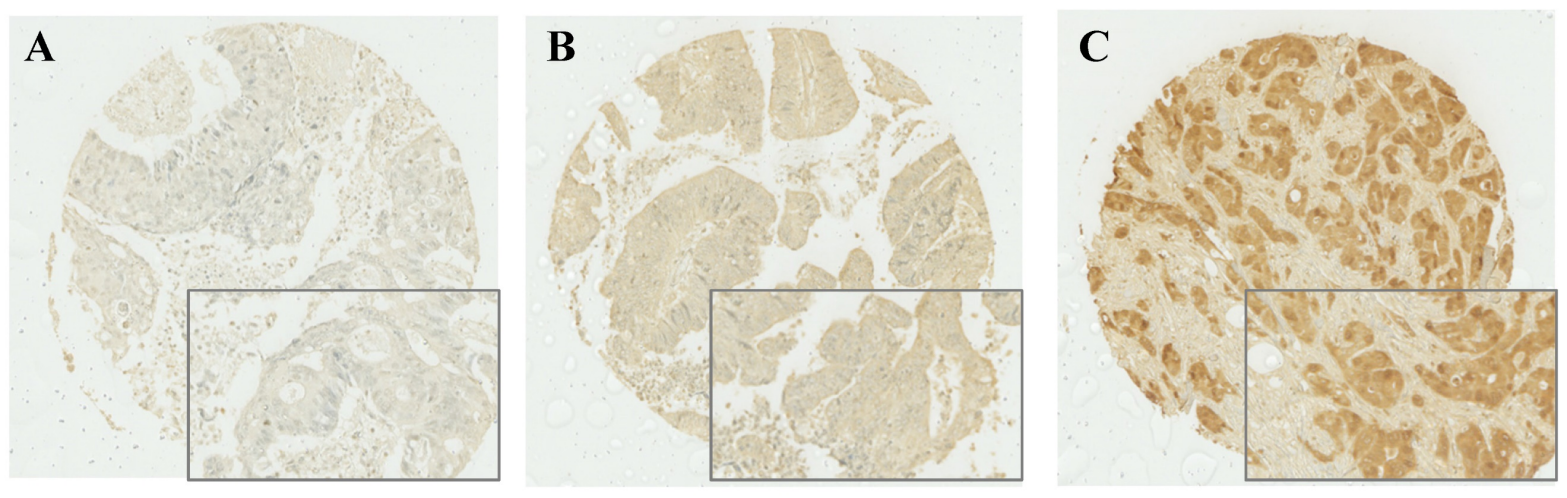
Cytoplasmic S100A2 staining; Weak (A), Medium (B), and Strong (C) expression in CRC TMAs.

After IHC was performed, cytoplasmic expression of S100A2 was observed and a weighted histoscore was employed to quantify protein expression (Figure 1). To classify patients into high and low expression of S100A2, a threshold of 90 was generated based on the histoscore of S100A2 using R packages. Patients were grouped according to weighted histoscore, those who has scored more than 90 were classified as having high expression and those with a lower or equal to 90 were classified as having low cytoplasmic S100A2 (Figure S4). The number of each group was obtained after the threshold has been applied. 442 patients classified as having high cytoplasmic S100A2 expression described as “high (n = 442)” and 177 patients were classified as having low cytoplasmic S100A2 expression described as “low (n = 177)”.

### S100A2 protein expression is associated with patient survival and clinicopathological factors

**Figure 2:**
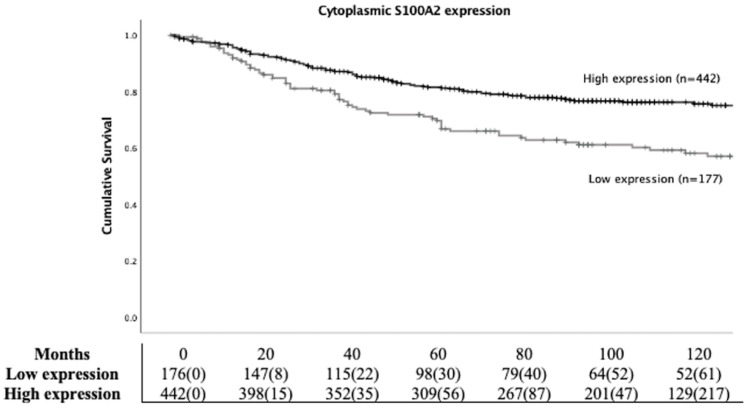
Kaplan-Meier survival analysis based on cytoplasmic S100A2 expression for cancer specific survival (CSS) in CRC patients.

To study the prognostic role of S100A2 in CRC, Kaplan Meier (KM) survival analysis was employed. Patients with high cytoplasmic S100A2 had significantly higher CSS compared to those groups with low cytoplasmic S100A2 (HR 0.539, 95%CI 0.394-0.737, P < 0.001) (Figure 2). Life tables demonstrated that 63% (112/177) patients with low S100A2 versus 78% (345/442) of patients with high S100A2 were alive at 5 years after initial diagnosis. S100A2 was then entered into Cox regression analysis. In univariate analysis, cytoplasmic S100A2 was associated with CSS, however, in multivariate analysis, it was not independent of the known clinical pathological parameters (Table S3).

The correlation between cytoplasmic S100A2 and the clinical characteristics of CRC patients is shown in Table 1. Chi-square test showed a significant association between low cytoplasmic expression of S100A2 and adverse clinical factors such as TNM (TNMIII, *P* < 0.001), T (T4, *P* = 0.009) and N (N1, *P*= 0.003) stages, local and distant recurrence (positive, *P* = 0.027 and *P*=0.034 respectively), peritoneal involvement (positive, *P* = 0.013), perineural invasion (positive, *P* < 0.001), Ki67 (low expression, *P* < 0.001), modified Glasgow prognostic score (mGPS) (mGPS1, *P* = 0.002).

### Gene expression from TempOSeq data

**Figure 3:**
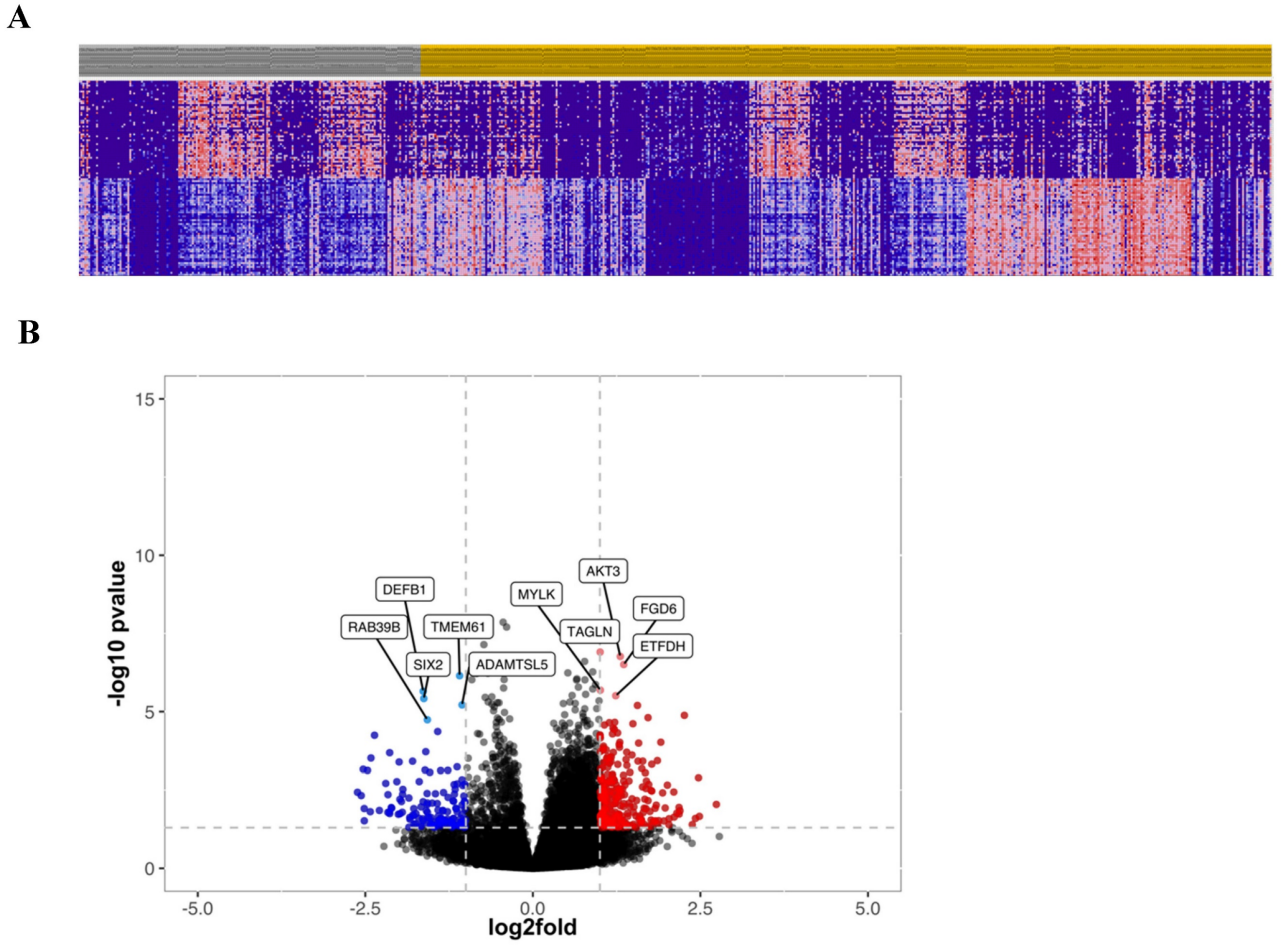
(A) Hierarchical clustering of the top 50 most differentially expressed genes between low (Grey) and high (Amber) S100A2 protein expression. (B) Volcano plot showing the distribution of gene expression fold changes and p values between patients with high and low S100A2; Red means up-regulated in cases with low S100A2 and blue means up-regulated in cases with high S100A2.

To further investigate the prognostic relevance of CRC patients with high and low cytoplasmic S100A2, transcriptomic data obtained from FFPE colorectal tissue in the same cohort was utilised. RNA expression was determined using TempO-Seq technology and tumours classified as either low (n = 175) or high (n = 435) for the S100A2 protein expression were compared using R packages for clustering analysis. The results showed no obvious classification between two groups as illustrated by PCA plot (Figure S5). Hierarchical clustering analysis was used to generate a heatmap for the top 50 differentially expressed genes comparing tumour cases with low S100A2 to those with high S100A2 protein expression (Figure 3A). Volcano plot, analysed from DESeq2**,** demonstrated genes that were significantly differentially expressed in cases with low S100A2 compared to cases with high S100A2. Regarding the outcome of patients with low cytoplasmic S100A2 as showed in the above results (Figure 1), genes significantly overexpressed in low cytoplasmic S100A2 groups such as *AKT3*, *TAGLN*, *MYLK, FGD6* and *ETFDH* have been observed (Figure 3B).

### GSEA immune profile compared between tumour with low and high S100A2 expression

**Figure 4:**
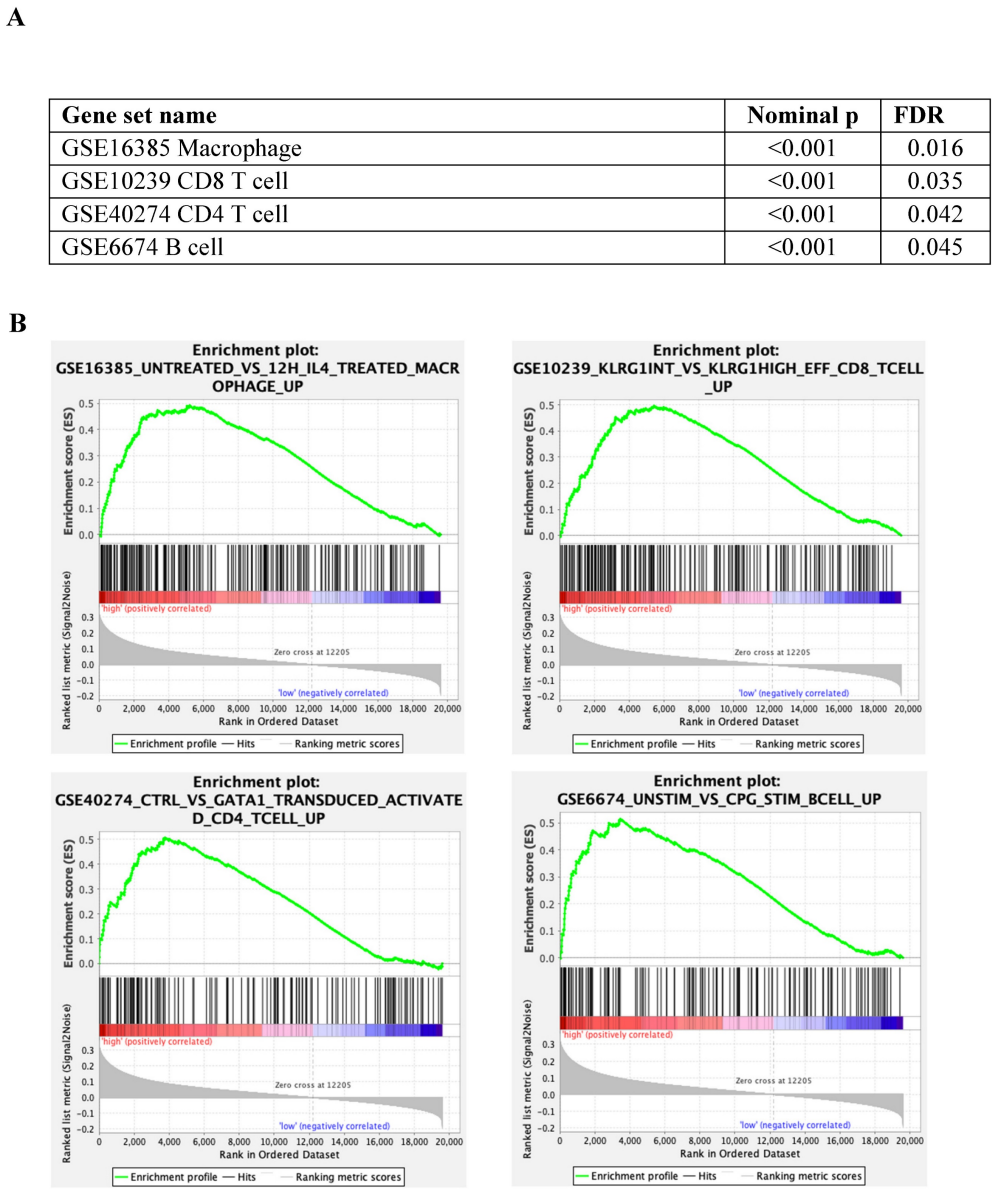
(A) Enrichment analysis by tumour with low and high cytoplasmic S100A2 in CRC from GSEA software (B) Enrichment plots using gene set immune signature (c7.all.v2022.1.Hs.symbols.gmt database).

The immunes signature gene set database was utilised to identify the different immunogenic patterns between tumours with low and high cytoplasmic S100A2 (Figure 4A). According to the analysis, up-regulated genes related to macrophages (nominal p < 0.001, FDR = 0.016) , CD8 T cells (nominal p < 0.001, FDR = 0.035), CD4 T cells (nominal p < 0.001, FDR = 0.042) and B cells (nominal p < 0.001, FDR = 0.045), and was enriched in tumour with high cytoplasmic when compared to low cytoplasmic S100A2 (Figure 4B).

### Relationship between S100A2 and immune cells protein expression

**Figure 5:**
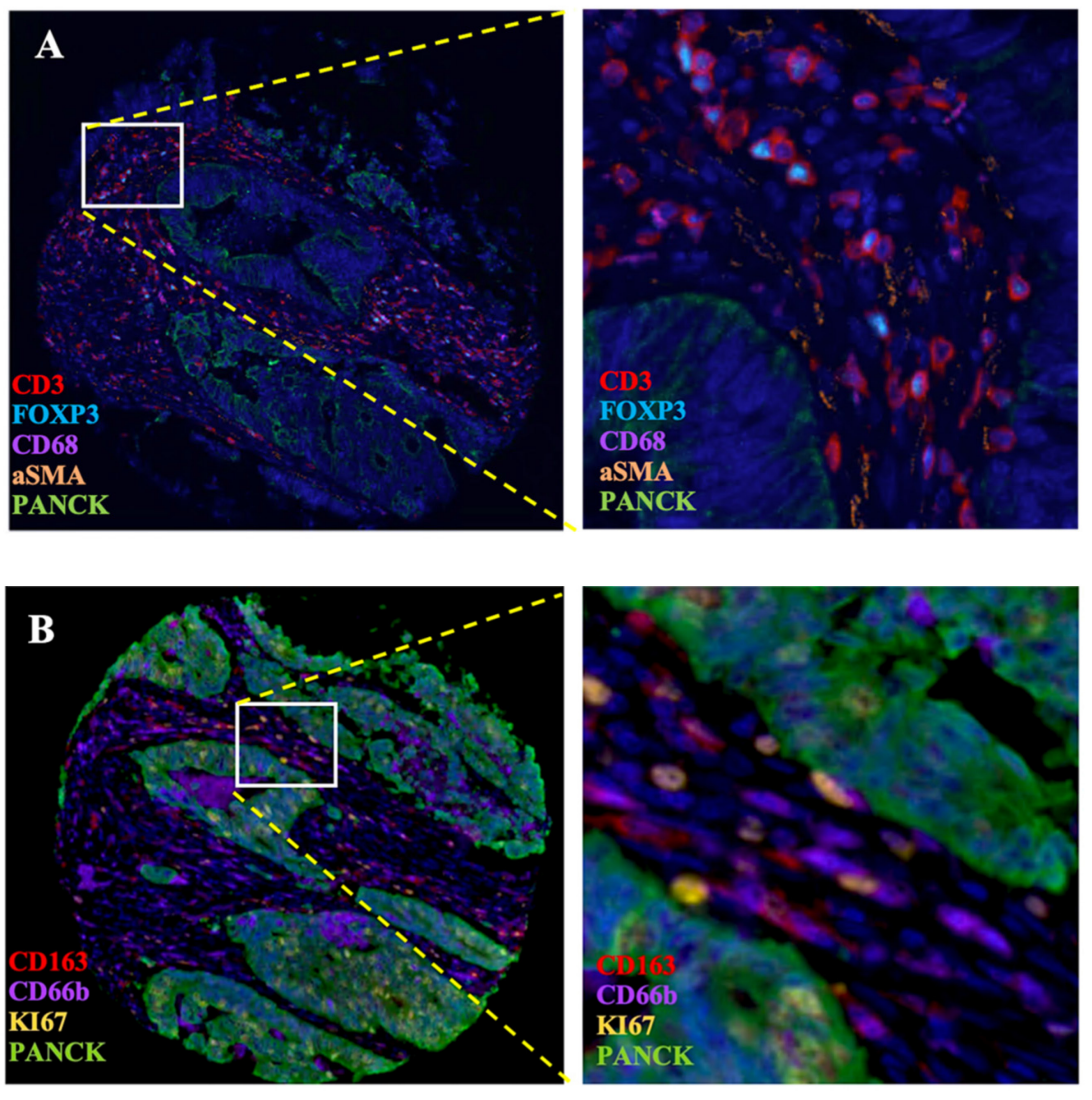
Multiplex staining of immune markers in CRC TMAs. (A) Panel of CD3, FOXP3, CD68, aSMA and PanCK and (B) Panel of CD163, CD66b, KI67 and PanCK. Magnification 4.87X. Magnification for zoom-in image 20X. Images generate on Visiopharm.

**Figure 6:**
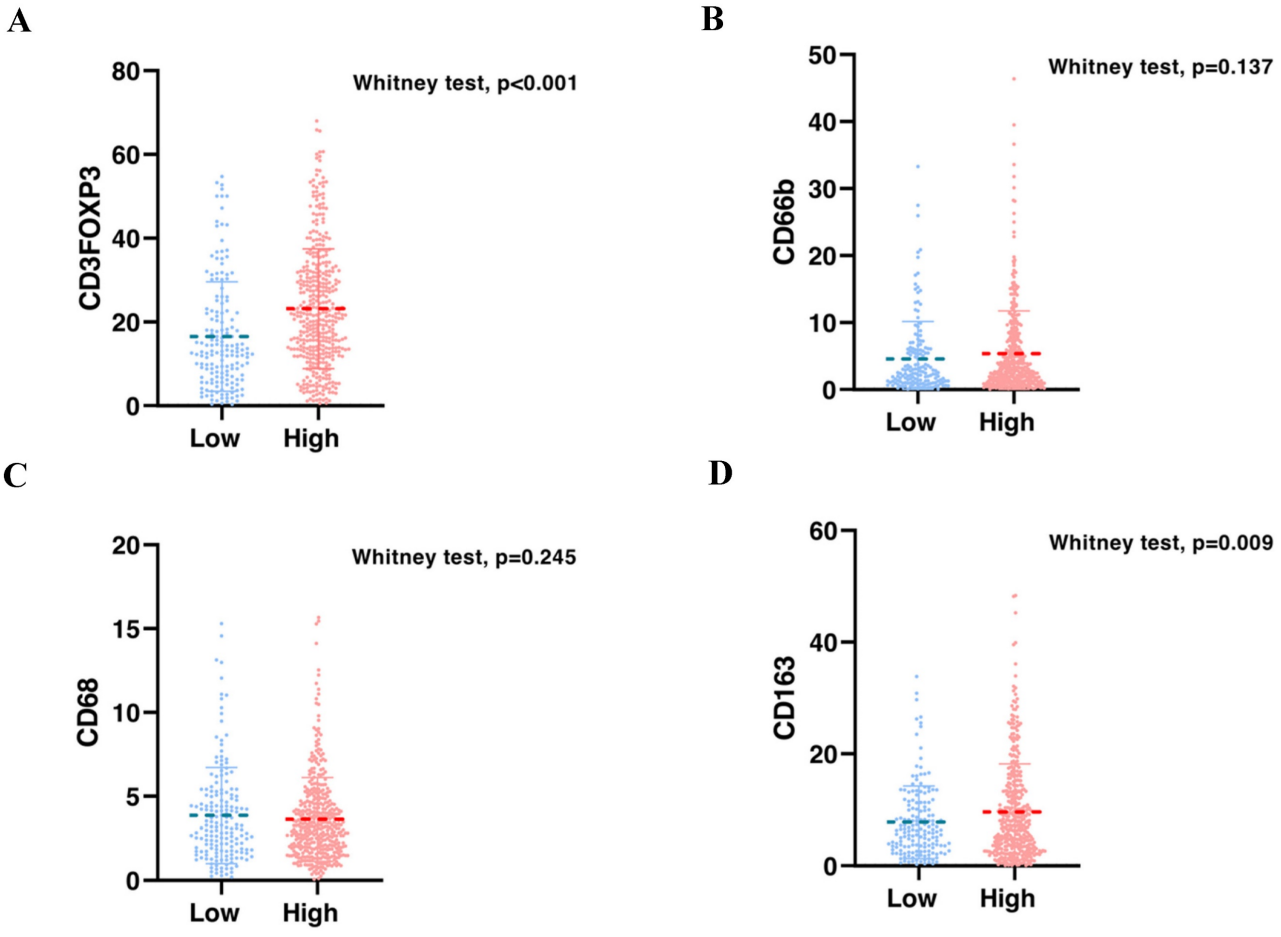
Bar charts showing the percentage of (A) CD3FOXP3 and (B) CD66b and (C) CD68 and (D) CD163 relative to S100A2 status in CRC.

To validate the correlation between S100A2 and immune cells shown at the mRNA level. Using the CRC TMA (n = 787) to stain for multiplex immunohistochemistry, the panel of immune cells; T lymphocyte (CD3, FOXP3), macrophages (CD68, CD163) and granulocyte (CD66b) were stained together with αSMA and PanCK for tissue segmentation (Figure 5). The results were analysed using image analysis to obtain the normalised count per core. Cut point, using R packages, was use to define high and low infiltrated phenotypes (Figure S6). The results demonstrated that tumours with high cytoplasmic S100A2 were enriched for infiltration of CD3+FOXP3+ cells (positive, *P* < 0.001) and CD163+ cells (positive, P = 0.009). There was no significant correlation found when S100A2 expression was assessed for association with CD68+ and CD66b+ cells (Figure 6A-D).

## Discussion

The prognostic role of S100A2 in CRC remains unclear. To understand the role of S100A2 in our CRC cohort, protein expression performed by IHC staining of S100A2 in TMAs of tumour core was observed. According to our findings, patients with high cytoplasmic S100A2 expression experienced a significant increase in survival when compared to patients with low cytoplasmic S100A2 expression, therefore low expression was associated with poor prognosis. In line with this observation, low protein expression of S100A2 was associated with adverse clinicopathological factors. Similar results were reported in gastric cancer, loss S100A2 expression was significantly associated with poor prognosis and shown to be an independent predictor using multivariate analysis 10. Recently, the study showed that mRNA expression of S100A2 is higher in CRC than those in normal colon tissue, and that level of S100A2 was associated with poor disease-free survival (DFS) of patients with CRC 28. In contrast with our finding, Masuda et al. reported that high S100A2 expression is associated with poor prognosis in CRC (n = 161) 12. Having said that, that was a much smaller cohort, with only CRC stage II and III, than used in this current investigation and a different antibody was used for IHC staining. According to Han et al, nuclear S100A2 expression has shown a poor outcome when compared to cytoplasmic expression in the CRC cohort (n = 278) and that expression of S100A2 at the invasive front is higher compared to the tumour core in CRC tissue 29. In the current study, we can only observe S100A2 cytoplasmic expression from the TMAs tumour core of CRC patients, therefore a nuclear expression of S100A2 should also be investigated in both tumour core and invasive front to understand the role of S100A2 regarding its localisation in tumour cells. In addition, our results showed that S100A2 has a positive correlation with KI67, a proliferation marker, which concordance with a previous study in CRC cells that overexpression of S100A2 can promote proliferation via glycolysis through GLUT1 expression 30.

**Figure 7:**
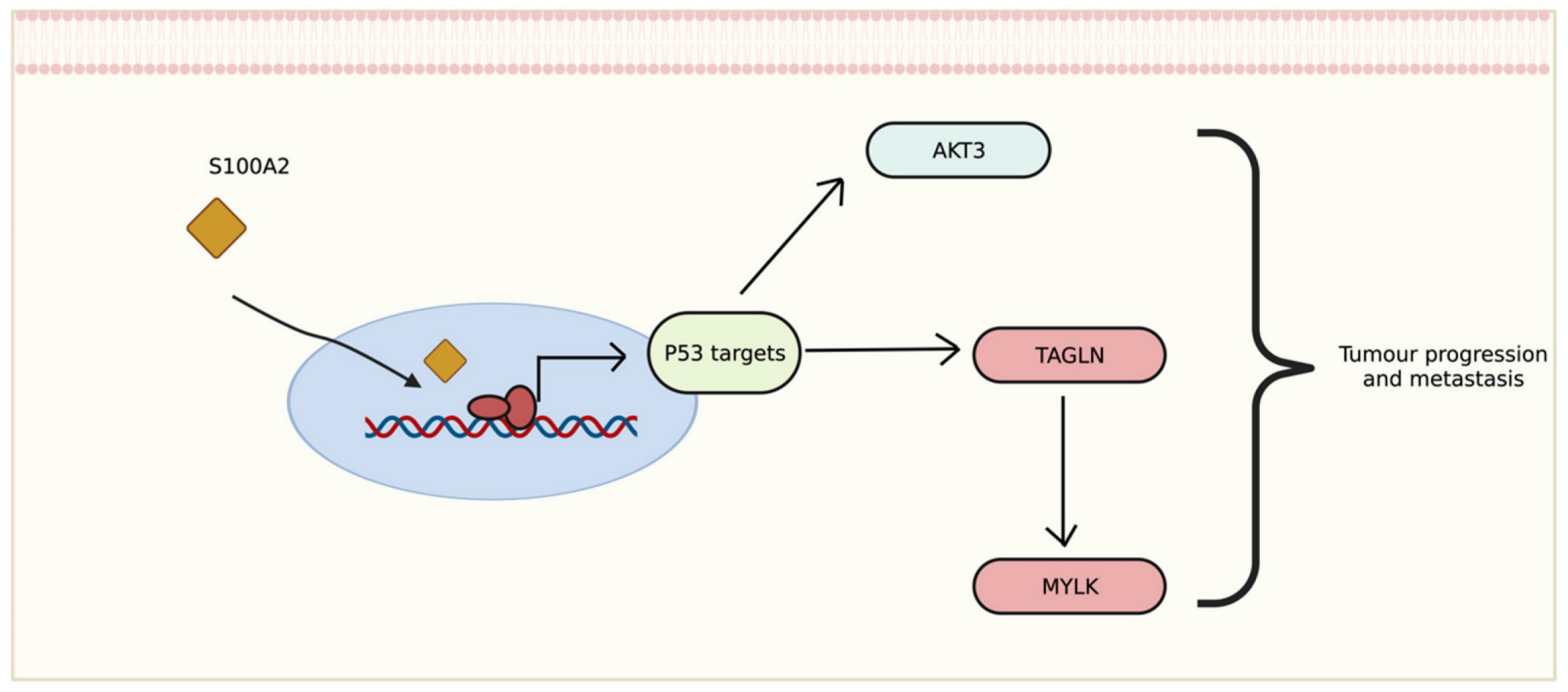
Proposed mechanism of S100A2 for regulating P53 to induce tumour-related signalling, created with BioRender.

The transcriptomic data showed that genes differentially expressed in tumour with low cytoplasmic S100A2 groups may correlate with disease development and progression. Interestingly, the correlation of up-regulated genes seem to be involved with p53 activation. The relationship between S100A2 and p53 also revealed a possible impact on cancer cell proliferation such as oral cancer 31, head and neck squamous carcinoma 32 and breast cancer 33. The upregulation of *AKT3*, one of the downstream signaliing of AKT pathway, has been shown to promote p53 regulation 34. This could perharps modulate AKT3 then further stimulate the signalling for tumour to progress (Figure 7). Morevoer, *TAGLN* has also been reported as a p53-upregulated gene in bladder cancer 35. The role of *TAGLN* in CRC is vaguely describe, though one study showed that it could bind to PARP1 which involved in Rho signalling, therefore, inducing metastasis of colon cancer cells 36. Eventhough there is no correlation with S100A2, upregulation of FGD6 have been reported as an independent factor to predict survival in patients with gastric cancer 37.

The role of S100A2 and inflammatory cells during cancer development is rarely reported 20, 38. Studies in pancreatic cancer have demonstrated that S100A2 could be used as a prognostic marker to identify patients who are more responsive to immunotherapy 16. According to our findings, the correlation between S100A2 expression and mGPS, a systemic inflammation-based score in our CRC cohort, can confirm that S100A2 may be involved in the inflammatory response and could be used as a potential biomarker to predict the efficiency of treatment in CRC patients 39. mRNA expression from GSEA also showed a upregulation of genes correlated with T cells, B cells and macrophages, in a group of patients with high cytoplasmic S100A2 suggesting a possible role of S100A2 in the immune response in CRC. To further validate the mRNA expression, multiplex staining with T lymphocyte (CD3, FOXP3), macrophages (CD68, CD163) and granulocyte (CD66b) markers was performed using TMAs of the same CRC cohort. In line with the mRNA expression, high tumoural cytoplasmic S100A2 expression was significantly associated with an increase number of CD3+FOXP3+ cells as higher influx of T lymphocytes indicates a favourable prognosis in CRC and that high cytoplasmic S100A2 showed a better outcome in our cohort, however there is conflicting evidence regarding the influence of the regulatory subset of T cells in CRC 40-42. The role of macrophages has been extensively published in CRC. The increased infiltration of M2-polarised macrophages together with the M1-polarised phenotype showed a better prognosis in patients with CRC 43. In parallel, we found a positive relationship with CD163 M2-like macrophages suggesting the contribution of immune activation when tumours have higher S100A2 expression. Additionally, the study using integrative-omics analysis revealed that high S100A2 expression was frequently observed when KRAS was mutated, which has been known to induce a series of inflammatory cells 44, compared to BRAF mutated CRC cells line 45. Therefore, future studies should be conducted to investigate both the genetic profile and the immunomodulatory functions of S100A2, especially in CRC patients.

## Conclusion

Low cytoplasmic S100A2 may have prognostic value in a patient's survival and is significantly associated with tumour-relating signaling and infiltrating immune cells. The role of S100A2 in CRC remains unclear and the underlying mechanisms of how it could be involved in disease development merit further study. Significant genes from transcriptomic data, associated with tumour with low cytoplasmic S100A2, could exhibit the prognostic role in CRC and the relationship between S100A2 and immune infiltration in CRC could inform future CRC treatment which warrants further study.

## Supplementary Material

Supplementary figures and tables.

## Figures and Tables

**Table 1:** The relationship between cytoplasmic S100A2 status and clinical characteristic in CRC patients

	Low expression of S100A2 N=177 (%)	High expression of S100A2 N=442 (%)	*P* value
**Host characteristics**			
**Sex**			0.197
Female	72 (41)	205 (47)	
Male	105 (59)	237 (53)	
**Age**			0.557
<65	50 (28)	144 (33)	
65-74	61 (35)	139 (31)	
>75	66 (37)	159 (36)	
**Tumour characteristic**			
**MMR status**			0.063
dMMR	40 (23)	67 (15)	
pMMR	105 (60)	300 (69)	
**Tumour site**			0.709
Right	70 (40)	182 (41)	
Left	107 (60)	260 (59)	
Local recurrence*			0.027
No	140 (86)	387 (92)	
Yes	22 (14)	32 (8)	
Distant recurrence*			0.034
No	119 (74)	342 (81)	
Yes	43 (43)	78 (19)	
**TNM stage**			<0.001
I	11 (7)	72 (16)	
II	79 (45)	217 (49)	
III	87 (49)	153 (35)	
**T stage**			0.009
1	5 (3)	22 (5)	
2	12 (7)	59 (13)	
3	101 (57)	260 (59)	
4	59 (33)	101 (23)	
**N stage**			0.003
0	90 (51)	289 (65)	
1	62 (35)	112 (25)	
2	25 (14)	41 (9)	
**Margin involvement**			0.159
No	163 (92)	420 (95)	
Yes	14 (8)	22 (5)	
**Peritoneal involvement**			0.013
No	125 (71)	353 (80)	
Yes	52 (29)	89 (20)	
Perineural invasion*			<0.001
No	44 (61)	193 (85)	
Yes	28 (39)	35 (15)	
**Tumour perforation**			0.718
No	173 (98)	434 (98)	
Yes	4 (2)	8 (2)	
**Venous invasion**			0.394
No	92 (52)	213 (48)	
Yes	85 (48)	229 (52)	
**TB^a^**			0.619
Low	125 (72)	297 (70)	
High	48 (28)	126 (30)	
**KI67^a^**			<0.001
Low	71 (51)	104 (27)	
High	69 (49)	285 (73)	
**Tumour microenvironment**			
TSP^*^			0.080
Low	129 (74)	340 (80)	
High	46 (26)	84 (20)	
KM^*^			0.357
Low	149 (86)	350 (83)	
High	25 (14)	74 (17)	
GMS^*^			0.129
0	25 (14)	74 (17)	
1	106 (61)	275 (65)	
2	43 (25)	75 (18)	
**mGPS**			0.002
0	101 (57)	292 (66)	
1	54 (31)	79 (18)	
2	22 (12)	71 (16)	

dMMR = deficient mismatch repair, pMMR = proficient mismatch repair, TB =Tumour budding, TSP = Tumour stroma percentage, KM = Klintrup-Mäkinen, GMS = Glasgow Microenvironment Score, mGPS = modified Glasgow prognostic score * some data missing from clinical record ^a^ some data not available

## Abbreviations

S100A2: S100 calcium-binding protein A2
CRC: colorectal cancer
IHC: immunohistochemistry
TMAs: tissue microarrays
TNF-α: Tumour necrosis factor alpha
mIF: multiplex immunofluorescence
PVDF: polyvinylidene fluoride
TBST: Tris-buffered saline with Tween
FFPE: formalin-fixed paraffin-embedded
TempO-Seq: Templated Oligo-Sequencing
MSigDB: molecular signature database
GSEA: Gene set enrichment analysis
HR: Hazard ratio
CSS: Cancer-specific survival
FDR: false discovery rate
CI: Confidence interval
