# Supplementary material for: Protein expression of S100A2 reveals it association with patient prognosis and immune infiltration profile in colorectal cancer
Source: J Cancer. 2023 Jun 19;14(10):1837–47. doi: 10.7150/jca.83910 (PMC10355195; doi:10.7150/jca.83910)
Supplement: Supplementary file 1 — Supplementary figures and tables. [file jcav14p1837s1.pdf]

Supplementary figures

$$\frac{\text{Total } \textbf{number of cells} \text{ count in stroma}}{\text{Total cells count in stroma}} \times 100 = \text{Percentage of cells on stroma area}$$

FigureS1 To calculate the percentage of positive cells in the stromal area, 3 replicates TMAs were constructed and obtained the average value of the staining. After that, the averaged value of positive cells was divided by the total number of cells to acquire the percent proportion of positive cells in each TMA core either in tumour or stromal area.

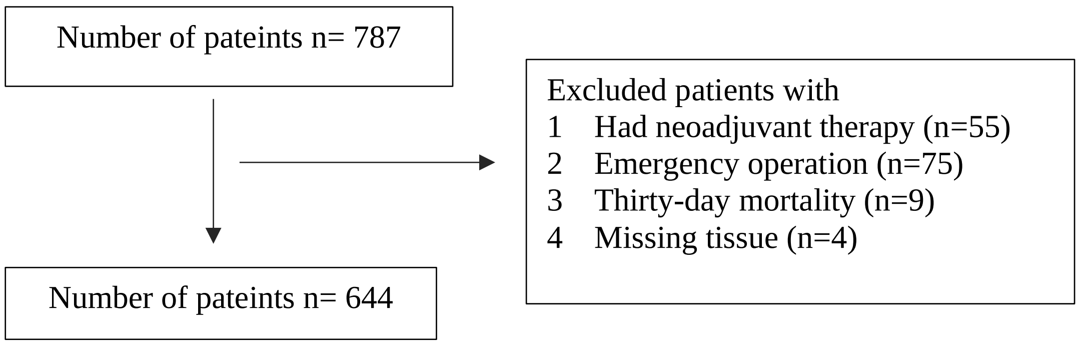

FigureS2 Consort diagram demonstrating how patients have been selected for the study

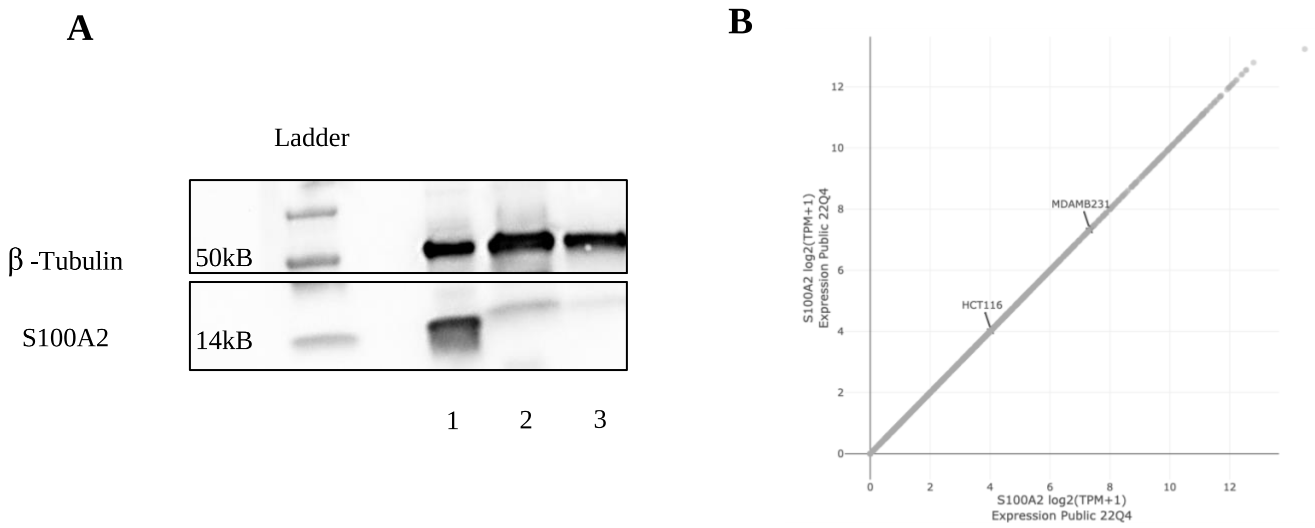

FigureS3 Antibody specificity test (A) Protein expression of S100A2 using western blot analysis of (1) 293T overexpressed S100A2 , (2) MDA-MB-231 and (3) HCT116 protein lysates. Precision Plus Protein^{TM} Dual Xtra Prestained Protein Standards (Bio-Rad) was used to visualised molecular weight. Samples were load onto for antibody specificity (n=3). S100A2 was 14 kB and β-tubulin were used for internal housekeeping control (50 kB) (B) S100A2 expression in tumour cell lines from DepMap

CSS Cut Offs

	cutpoint	statistic
**S100A2_N**	90	2.726030589344

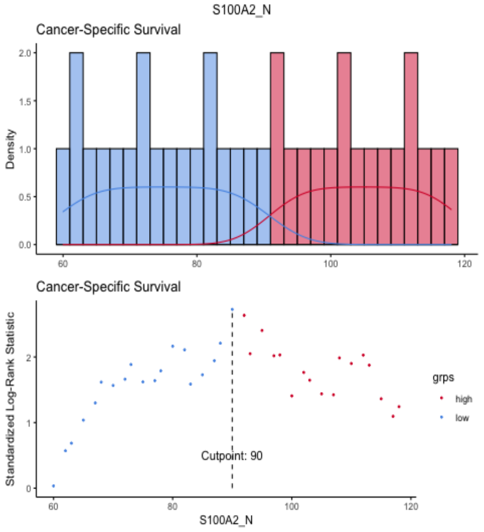

FigureS4 Cut point generated from R program defined low and high expression of cytoplasmic S100A2 with cancer specific survival

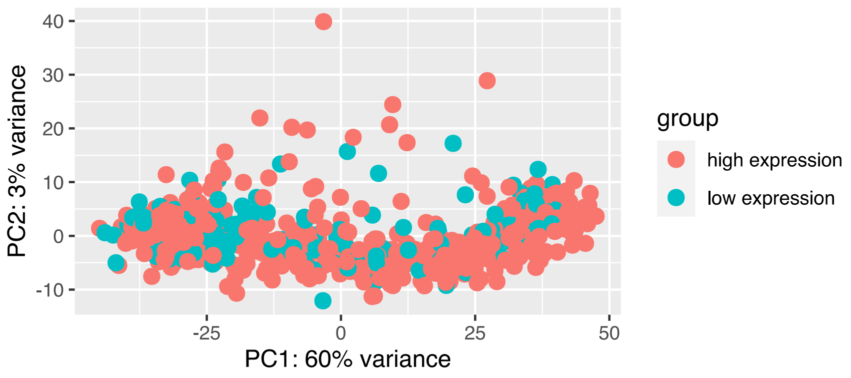

FigureS5 PCA plot showed the clustering between high (red) and low (blue) cytoplasmic S100A2

CSS cut offs		
	**cutpoint**	**statistic**
**CD3FOXP3**	15	1.94
**CD68**	8.24	4.57
**CD163**	7.35	1.73
**CD66b**	1.21	3.48
**KI67**	3.27	3.65

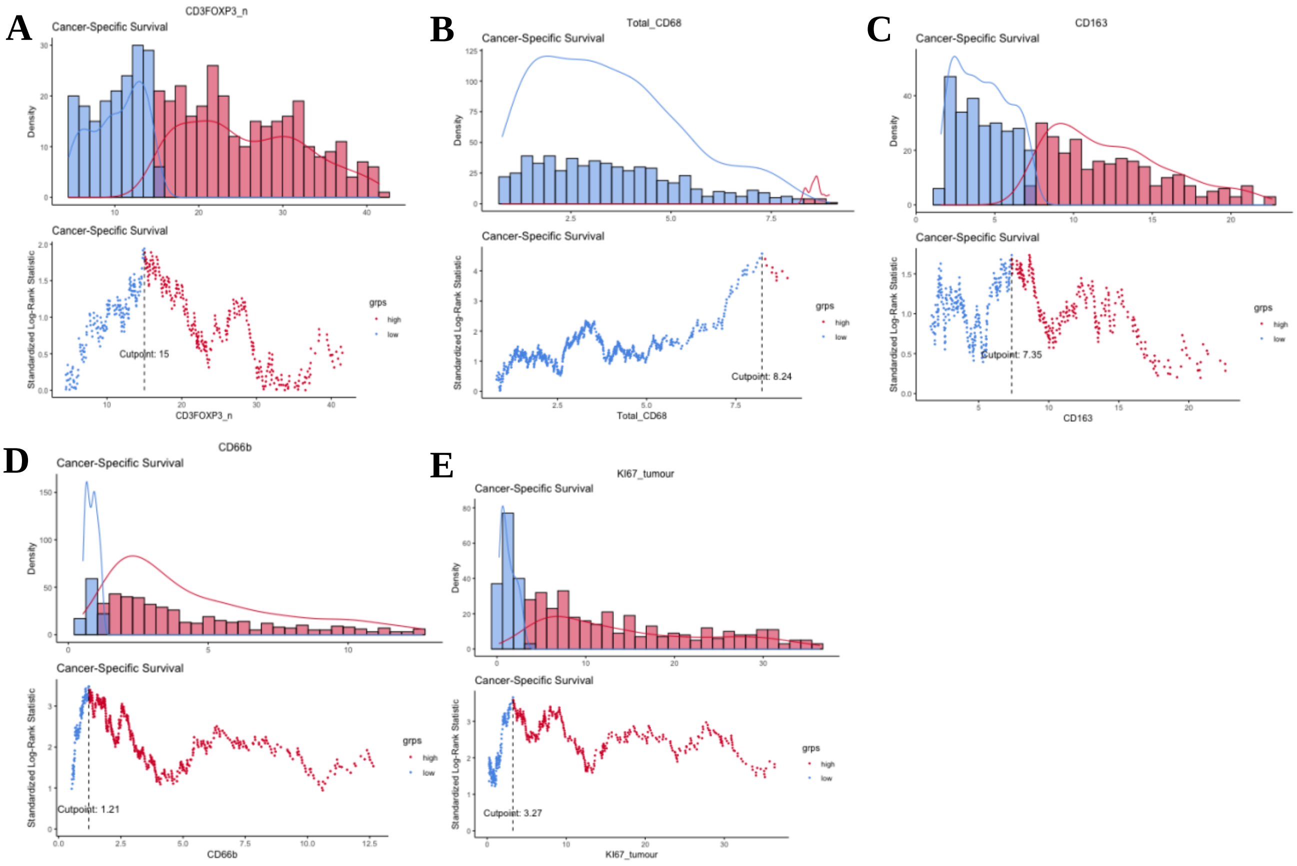

FigureS6 Cut point generated from R program defined low and high expression of (A) CDFOXP3 (B) CD68 (C) CD163 (D) CD66b and (E) KI67 with cancer specific survival

Supplementary tables

TableS1 Antibodies or multiplex staining

**Antibody**	**Clone**	**Company**	**Dilution**
anti-CD3 (2GV6)	Rabbit Monoclonal Primary Antibody	Roche Tissue Diagnostics (790-4341)	RTU
Pan-Cytokeratin (AE1/AE3	Mouse Monoclonal Antibody	Leica Biosystems (AE1/AE3-601-L-CE)	1:250
FOXP3 (236A/E7)	Mouse Monoclonal Antibody	abcam (ab20034)	1:20
Actin, Smooth Muscle (1A4)	Mouse Monoclonal Antibody	Roche Tissue Diagnostics (760-2833)	RTU
CD8a (C8/144B)	Mouse Monoclonal Antibody	Cell Signaling Technology (70306)	1:100
CD68 (D4B9C) XP®	Rabbit Monoclonal Antibody	Cell Signaling Technology (76437)	1:200
CD163	Rabbit Monoclonal Antibody	Abcam (ab182422)	1:45000
CD66b	Mouse Monoclonal Antibody	Novus Biological (NB100-77808)	1:8000

RTU =Ready to use

Table S2 Patients characteristic (N=644)

Characteristic		
Sex		
	Female	291 (45%)
	Male	353 (55%)
Age		
	<65	205 (32%)
	65-74	208 (32%)
	>75	231 (36%)
Tumour site		
	Right	260 (40%)
	Left	384 (60%)
MMR status		
	dMMR	111 (17%)
	pMMR	418 (65%)
	NA	100 (16%)
	Missing data	15 (2%)
Local recurrence		
	No	551 (86%)
	Yes	55 (9%)
	Missing data	38 (5%)
Distant recurrence		
	No	480 (75%)
	Yes	127 (20%)
	Missing data	37 (5%)
TNM stage		
	I	87 (14%)
	II	308 (48%)
	III	249 (39%)
T stage		
	1	30 (5%)
	2	74 (12%)
	3	375 (58%)
	4	165 (25%)
N stage		
	0	395 (61%)
	1	182 (28%)
	2	67 (10%)
Margin involvement		
	No	608 (94%)
	Yes	36 (6%)
Peritoneal involvement		
	No	498 (77%)
	Yes	146 (23%)

Tumour perforation		
	No	631 (98%)
	Yes	13 (2%)
Venous invasion		
	No	320 (50%)
	Yes	324 (50%)
TB		
	Low	441 (69%)
	High	180 (28%)
	Missing data	23 (3%)
TSP		
	Low	490 (76%)
	High	133 (21%)
	Missing data	21 (3%)
KM		
	Low	519 (81%)
	High	103 (16%)
	Missing data	22 (3%)
GMS		
	0	103 (16%)
	1	399 (62%)
	2	120 (19%)
	Missing data	22 (3%)
mGPS		
	0	409 (64%)
	1	137 (21%)
	2	98 (15%)

dMMR = deficient mismatch repair, pMMR = proficient mismatch repair, TB =Tumour budding, TSP = Tumour stroma percentage, KM = Klintrup-Mäkinen, GMS = Glasgow Microenvironment Score, mGPS = modified Glasgow prognostic score, NA= not available

Clinicopathological characteristics	Univariable analysis		Multivariate analysis	
	**Hazard ratio (95% CI)**	***P***	**Hazard ratio (95% CI)**	***P***
**Sex**	1.32 (0.97-1.79)	0.082		
**Age**	1.29 (1.06-1.55)	0.009	1.40 (1.13-1.75)	0.003
**MMR status**	0.67 (0.52-0.88)	0.003		
**Local recurrence**	6.95 (4.85-9.98)	<0.001	3.83 (2.60-5.64)	<0.001
**Distant recurrence**	24.39 (16.26-36.58)	<0.001	23.14 (14.76-36.27)	<0.001
**TNM stage**	2.49 (1.92-3.24)	<0.001		
**T stage**	1.88 (1.49-2.38)	<0.001		
**N stage**	2.09 (1.72-2.54)	<0.001	1.64 (1.28-2.12)	<0.001
**Margin involvement**	4.05 (2.58-6.34)	<0.001	3.67 (2.01-6.67)	<0.001
**Peritoneal involvement**	2.07 (1.51-2.84)	<0.001		
**Tumour perforation**	2.65 (1.17-5.99)	0.019		
**Venous invasion**	1.51 (1.11-2.04)	0.008		
**TB**	1.88 (1.37-2.56)	<0.001	1.68 (1.16-2.42)	0.006
**S100A2**	0.54 (3.94-0.74)	<0.001		
**GMS**	1.74 (1.35-2.25)	<0.001		
**mGPS**	1.42 (1.18-1.72)	<0.001		

Table S3 Univariate and Multivariate analysed for cancer specific survival (CSS)
MMR = Mismatch repair, TB = Tumour budding, GMS = Glasgow Microenvironment Scores, mGPS = modified Glasgow prognostic score
